# Selective single-atom electrocatalysts: a review with a focus on metal-doped covalent triazine frameworks

**DOI:** 10.1039/d0sc03328f

**Published:** 2020-07-20

**Authors:** Kazuhide Kamiya

**Affiliations:** Research Center for Solar Energy Chemistry, Osaka University 1-3 Machikaneyama Toyonaka Osaka 560-8531 Japan kamiya@chem.es.osaka-u.ac.jp; Graduate School of Engineering Science, Osaka University 1-3 Machikaneyama Toyonaka Osaka 560-8531 Japan; Japan Science and Technology Agency (JST) PRESTO 4-1-8 Honcho Kawaguchi Saitama 332-0012 Japan

## Abstract

Single-atom electrocatalysts (SACs), which comprise singly isolated metal sites supported on heterogeneous substrates, have attracted considerable recent attention as next-generation electrocatalysts for various key reactions from the viewpoint of the environment and energy. Not only electrocatalytic activity but also selectivity can be precisely tuned *via* the construction of SACs with a defined coordination structure, such as homogeneous organometallics. Covalent organic frameworks (COFs) are promising supports for single-atom sites with designed coordination environments due to their unique physicochemical properties, which include porous structures, robustness, a wide range of possible designs, and abundant heteroatoms to coordinate single-metal sites. The rigid frameworks of COFs can hold unstable single-metal atoms, such as coordinatively unsaturated sites or easily aggregated Pt-group metals, which exhibit unique electrocatalytic selectivity. This minireview summarizes recent advances in the selective reactions catalysed by SACs, mainly those supported on triazine-based COFs.

## Abstract 2

Single-atom electrocatalysts (SACs) have attracted considerable attention as selective electrocatalysts. Metal-doped covalent triazine frameworks will be a novel platform for selective SACs to solve energy and environmental issues.

## Graphical abstract

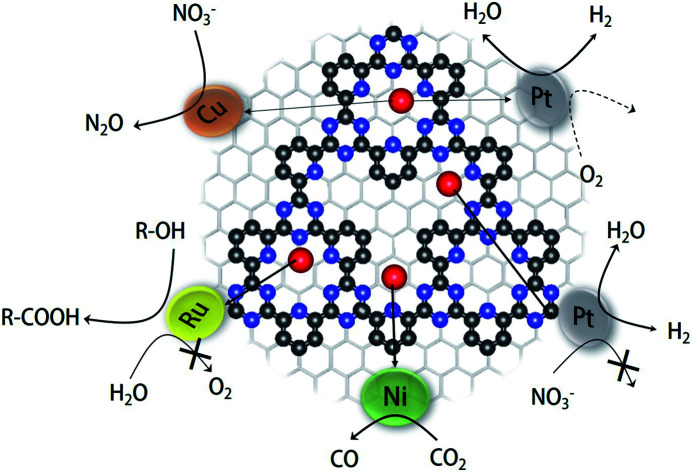

## Introduction

Electrochemical devices are attracting increasing attention in applications such as batteries,^1^ artificial photosynthesis,^2^ and pollutant purifiers^3–6^ because they can function under ambient conditions without toxic chemicals. Electrocatalysts are one of the most important components of electrochemical devices. Thus, the development of efficient electrocatalysts for reactions that solve energy and environmental problems, including the hydrogen evolution reaction (HER), oxygen reduction reaction (ORR),^7^ oxygen evolution reaction (OER),^8^ and carbon dioxide reduction reaction (CRR),^9,10^ is strongly desired.^11^

Although high reaction rates are essential for the efficient electrocatalysis of these reactions, selectivity should also be considered a fundamental requirement. Two kinds of selectivity are required in these electrochemical reactions (1) product selectivity (*e.g.*, various products from the CRR): and (2) substrate selectivity (*e.g.*, a methanol-tolerant ORR).^12,13^ The control of product selectivity toward high-value-added chemicals is essential because product purification can be drastically simplified, enhancing the technological competitiveness of electrolysis. On the other hand, improving substrate selectivity enables the utilization of contaminated (*i.e.*, low-purity) substrates, which leads to an expansion of electrochemical methods as an on-demand technology. One conventional approach to obtaining selective electrocatalysts is to utilize dissolved organometallics or to physically or chemically immobilize them onto an electrode.^14–16^ The most important advantage of organometallics is that we can freely modulate the electronic and geometrical structures of their metal centres. The high design flexibility of organometallics leads to not only high activity but also high selectivity. However, the stability of these small organic compounds under electrochemical conditions remains inadequate.

Recently, single-atom electrocatalysts (SACs), which comprise singly isolated metal atoms immobilized onto a heterogeneous support, have attracted intensive attention as a robust analogue of organometallics.^17–24^ SACs are the ultimate form of size reduction of metal electrodes, and they maximize the efficiency of metal atom use. Furthermore, some SACs exhibit unique electrocatalytic activity and selectivity. Therefore, SACs are expected to be a new platform for heterogeneous metal-based electrocatalysts. The formation of a defined coordination structure of single metal atoms like organometallics is essential for practical SACs. SACs with defined coordination structures on inorganic supports, including nanocarbons, metal oxides, and nitrides, have recently been developed and are well summarized in reviews.^23,24^ For example, Kou and Wang *et al.* synthesized single Mo sites with an Mo_1_N_1_C_2_ local coordination structure in nitrogen-doped carbons and used them as efficient bifunctional OER/ORR and nitrogen reduction reaction catalysts.^25,26^ The same authors nicely demonstrated that the single Co atoms which are coordinated with three Mo atoms in the 2D molybdenum carbide nanosheets served as effective active sites for both the OER and the HER.^27^

An alternative approach to obtain SACs with a designed structure is to use heterogeneous organic supports with high design flexibility. Covalent organic frameworks (COFs), which are a class of conjugated microporous polymers, have attracted intensive attention as novel polymeric materials for use in heterogeneous catalysts and optical and electrical materials because of their unique physicochemical properties, which include nanoporous structures and chemical and mechanical robustness.^28–32^ COFs are expected to overcome the aforementioned problems with conventional SAC supports and to serve as the preferred platform for SAC supports for the following reasons: (1) they exhibit large surface areas because of their microporosity; (2) their structures can be designed with excellent flexibility through the choice of appropriate monomers; and (3) they can be prepared with abundant heteroatoms with a lone electron pair, such as N, S, and O, to strongly immobilize metals *via* coordination bonds. Therefore, COFs can be ideal materials for extending single-metal centres to heterogeneous catalysts while maintaining the wide range of possible designs of homogeneous organometallics.

In this minireview, we focus on the selectivity of SACs, mainly those supported on COFs. Although the literature contains several excellent reviews on the activity of SACs, it does not, to my knowledge, include a significant review focused on the selectivity of SACs. This review will therefore provide readers with important insights into and design strategies for selective electrocatalysts.

## Synthesis and characterization of single-atom-doped triazine-based COFs

### Synthesis of triazine-based COFs hybridized with conductive carbon nanoparticles

COFs can be roughly classified according to their linkages into three types: boron-, triazine-, and imine-based COFs.^30^ The present minireview focuses on triazine-based COFs (CTFs: covalent triazine frameworks) because, among the three classes of COFs, CTFs are the most stable in aqueous solution and under electrochemical conditions. CTFs were first prepared through ionothermal synthesis in 2008.^33,34^ Palkovits *et al.* successfully applied Pt-modified pyridine-linked CTFs as a catalyst for the partial oxidation of methane to methylbisulfate in concentrated sulfuric acid.^35^ Several studies on thermal catalysts composed of metal-modified CTFs followed.^36,37^ However, these CTFs exhibited poor electron conductivity; CTF-based materials were therefore not applied as electrocatalysts until 2014.

**Fig. 1:**
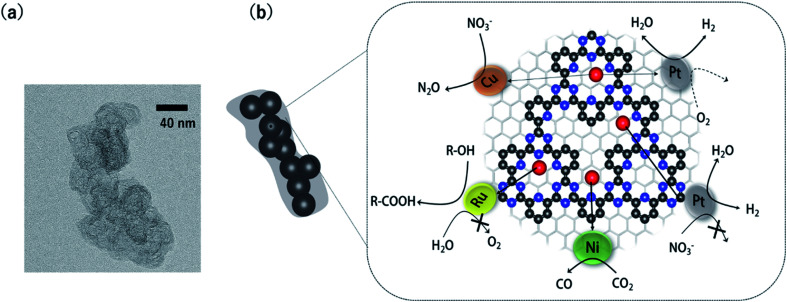
(a) A representative TEM image of the CTF/carbon nanoparticle hybrid (b) various selective electrocatalytic reactions catalysed by metal-doped CTFs hybridized with carbon nanoparticles (blue: N, red: single-metal atoms and black: C).

Our group overcame the poor conductivity problem by polymerizing CTFs from 2,6-dicyanopyridine onto conductive carbon nanoparticles (Fig. 1).^38^ In the transmission electron microscopy (TEM) image of the CTF/carbon particle hybrid, the approximately 40 nm particles correspond to the carbon nanoparticles. The obtained CTF hybridized with the carbon particles can immobilize various single-metal atoms through coordination bonds. These metal-doped CTFs (M-CTFs) exhibit unique electrocatalytic selectivity depending on the immobilised metal species, as reviewed in the next section.

### Characterization of single-metal atoms in M-COFs

**Fig. 2:**
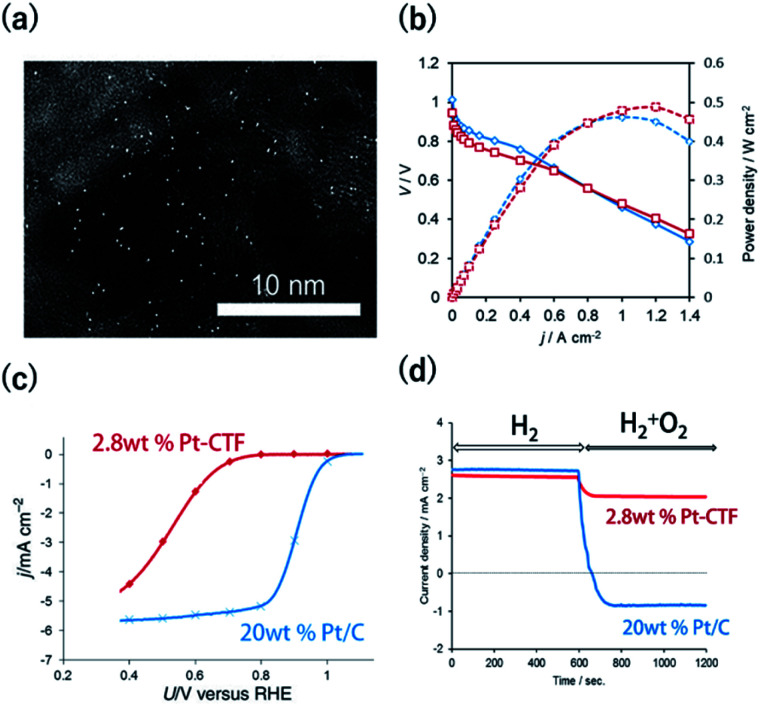
(a) A representative HAADF-STEM image of a Pt-CTF. (b) *j*–*V* curves (solid line) and *j*-power density curves (dashed line) for MEAs. Anode catalysts: (red) 2.8 wt% Pt-CTF (0.020 mg_-Pt_ cm^−2^) and (blue) 20 wt% Pt/C (0.10 mg_-Pt_ cm^−2^). (c) *j vs. U* curves for the ORR at a sweep rate of 10 mV s^−1^ and a rotation rate of 1600 rpm in 0.1 M HClO_4_ at 25 °C. Catalysts: (red) 2.8 wt% Pt-CTF, (blue) 20 wt% Pt/C. (d) Current *vs.* time curves for (red) 2.8 wt% Pt-CTF and (blue) 20 wt% Pt/C (0.6 V *vs.* RHE). The input gas was altered at 600 s from pure H_2_ to a H_2_/O_2_ mixed gas (H_2_ : O_2_ = 50 : 50). Reprinted in part with permission from ref. 46. Copyright 2016 John Wiley and Sons.

The methods to characterize single-metal atoms are briefly explained here. For more detailed information on the characterization of SACs, the reader is referred to the review literature.^18,19,23^ High-angle annular dark-field scanning transmission electron microscopy (HAADF-STEM) is a powerful method for discerning isolated heavy atoms (*e.g.*, Fig. 2a in the next section). The other important and basic tool for characterizing the coordination environments of SACs is extended X-ray absorption fine structure (EXAFS) analysis. Curve fitting of the Fourier transformation (FT)-EXAFS analysis results is a direct approach for identifying the coordination structure of SACs (*e.g.*, Fig. 4b). However, the critical drawback of EXAFS is that only the average spectra can be obtained. Recently, electron energy loss spectroscopy (EELS) and scanning tunnelling microscopy (STM) have been used to directly characterize single-atoms in SACs with atomic resolution.^26,27,39^ These methods will soon clarify the detailed coordination environments of M-COFs.

The importance of *in situ* and operando techniques is increasing because SACs might aggregate or change the structure during electrolysis. Electrochemical *in situ* X-ray absorption fine structure (XAFS) measurements have been used to characterize the Cu-doped sulfur-linked CTF during the ORR, and the change in the valence state was clearly observed.^40^*In situ* Fourier transform infrared (FTIR) spectroscopy and surface-enhanced Raman spectroscopy (SERS) are also useful methods that have been well established for conventional metal-based electrocatalysts. However, the information provided by these techniques is still based on average values.

In addition to experimental characterization, density functional theory (DFT) calculations are also an important approach to not only investigate the pathway of electrocatalytic reactions but to clarify the coordination structure of SACs. Stabilization energy analyses using DFT reveal the most stable/favourable configurations of SACs^26,41,42^ (refer to the later section about stability).

## Selective electrochemical reactions by single-atom-doped CTFs

### The hydrogen oxidation reaction in the presence of oxygen

The HER and the hydrogen oxidation reaction (HOR) are key reactions for artificial photosynthesis and polymer electrolyte fuel cells (PEFCs), respectively. The development of efficient electrocatalysts is required for the further implementation of these technologies. At present, Pt nanoparticles (Pt_nano_) are used. However, because Pt is scarce and expensive, reducing the loading of Pt is highly desirable. Furthermore, catalysts of Pt_nano_ are active toward not only the HOR and HER but also the ORR, which is unfavourable for this application. For example, in the case of water splitting, photoexcited electrons are quickly consumed by the reduction of O_2_ molecules generated from water oxidation on Pt_nano_.^43^ Even if Pt_nano_ are used as the anode catalyst, air enters the fuel chamber, and the ORR proceeds on its surface during the start-up of the PEFC, leading to oxidative decomposition of the carbon particles on the cathode.^44,45^ Therefore, O_2_-tolerant HOR/HER catalysts are highly sought. Single-Pt-atom electrocatalysts might satisfy the requirements of (1) reducing Pt atom usage and (2) selectively catalysing HOR/HER reactions over the ORR. For example, Kamai and Kamiya *et al.* synthesized Pt-modified CTFs (Pt-CTF) by impregnating CTFs with K_2_[PtCl_4_] solutions at 30 °C.^46^ High-resolution transmission electron microscopy (TEM) images and the corresponding HAADF-STEM images show bright white spots assignable to Pt atoms (diameters < 0.5 nm) uniformly dispersed on CTFs; almost no Pt_nano_ (sizes > 1 nm) were observed (Fig. 2a). EXAFS spectra also show that Pt atoms were singly isolated on the CTFs.^46^ The single Pt atoms exhibited clear HOR activity from 0 V *vs.* reversible hydrogen electrode (RHE) in an acidic liquid electrolyte. The Pt-CTF was then loaded onto membrane electrode assemblies (MEAs) and used as the anode catalyst in fuel cells under real operating conditions. Importantly, when the loading amount of Pt in the Pt-CTF was 2.8 wt%, the electrocatalytic HOR activity of the resulting electrode was comparable to that of commercial carbon-supported 20 wt% Pt/C catalysts (Fig. 2b). The efficient utilization of Pt atoms was maximized by reducing the particle size to singly isolated forms. This work was the first demonstration that the Pt-SAC exhibits HOR activity.^46^ Notably, the Pt-CTFs exhibited more efficient activity than Pt/C even for the HER, which is the reverse of the HOR.

A Pt-CTF was later used as a selective HOR catalyst against the ORR. For 20 wt% Pt/C, the ORR started to occur at approximately 1.06 V and was diffusion-limited at approximately 0.80 V (blue curve in Fig. 2c). By contrast, the electrocatalytic ORR activity of the Pt-CTF was much lower (red curve in Fig. 2c). Fig. 2d shows the change in current at 0.6 V *vs.* RHE under H_2_ conditions by the addition of O_2_ for the Pt-CTF and conventional Pt/C catalysts. The polarity of the current changed from positive to negative upon the addition of O_2_ on the Pt/C catalysts because the HOR was hidden by a large cathodic ORR current. By contrast, the Pt-CTF showed almost no change in HOR current with and without O_2_. These results indicate that the Pt-CTF showed high O_2_ tolerance.^46^ The lower ORR activity on the Pt-SACs than on Pt_bulk_ has been well documented. For example, Lee *et al.* demonstrated that a single Pt atom shows poor ORR activity and selectively catalyses H_2_O_2_ formation *via* a two-electron reaction using single-Pt-loaded titanium(iii) nitride or carbide (TiN or TiC, respectively).^47–49^ Choi *et al.* also showed that the single Pt atoms in sulfur-doped zeolite-templated carbons selectively produced H_2_O_2_ during the ORR.^50^ This selectivity is attributed to a lack of dissociative adsorption of oxygen molecules on Pt-SACs because of the lack of adjacent Pt sites.

### The oxygen reduction reaction in the presence of organic contaminants

Another desired selectivity for Pt-group electrocatalysts for various applications is the anti-poisoning effect against organic impurities. In particular, methanol-tolerant ORR activity is an essential selectivity for the cathode catalysts used in direct methanol fuel cells (DMFCs) because methanol crossover from the anode to the cathode is one of the main problems to be addressed.^51^ The adsorption of oxygenated species onto a Pt site next to a methanol absorption site is known to be required for methanol oxidation. Therefore, single-Pt-atom sites are expected to be inactive for methanol oxidation.

**Fig. 3:**
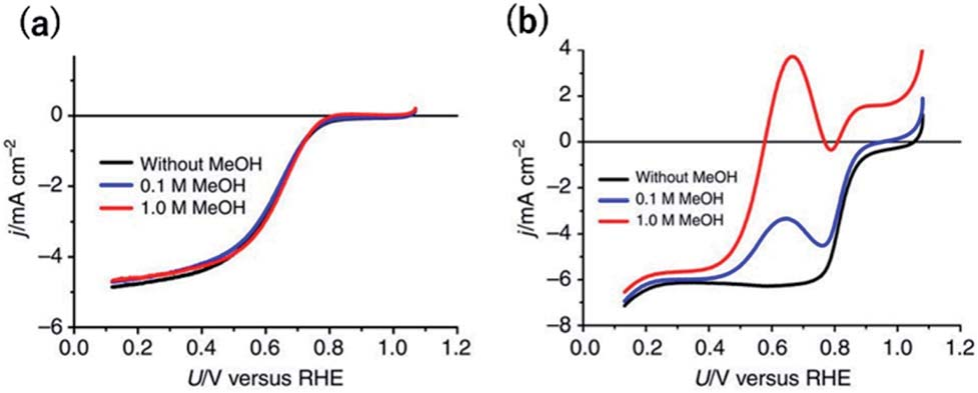
*j vs. U* curves for (a) 12 wt% Pt-CTF and (b) 20 wt% Pt/C in 0.5 M H_2_SO_4_ saturated with dissolved O_2_. The methanol concentration: (black) 0 M, (blue), 0.1 M and (red) 1 M. Reproduced from ref. 38 under the CC BY 4.0 license.

Although single-Pt-atom catalysts unfortunately show lower ORR activity than Pt_nano_, as mentioned in the previous section, methanol-tolerant ORR activity has been clearly observed. In the case of a commercial 20 wt% Pt/C, after the addition of 1 M methanol, the onset potential for the cathodic current became approximately 200 mV more negative (Fig. 3a).^38^ By contrast, surprisingly, the overlapped methanol oxidation current during the ORR was almost negligible for 12 wt% Pt-CTF even in the presence of 1 M methanol (Fig. 3b).^38^ Inactivity toward methanol oxidation has also been reported by Lee *et al.* for single Pt on TiC and TiN supports.^17,52,53^ They found that, in contrast to methanol oxidation, formic acid was effectively oxidized to CO_2_ on single Pt atoms. The reduced state of the Pt single atom enhanced the formic oxidation current. We here deeply discuss the mechanism of substrate selectivity toward the oxidation of organic compounds by a Pt-SAC. Methanol is oxidized on Pt electrodes *via* two pathways: an indirect pathway (or CO pathway) and a direct pathway (or non-CO pathway). For the indirect pathway, the dissociative adsorption of methanol onto an ensemble of empty Pt sites facilitates the C–H bond breaking to form CO.^54^ Even for the direct pathway, adsorbed oxygen species such as O_ad_ or OH_ad_ on the Pt site adjacent to the methanol adsorption site are required for the dehydrogenation of the O–H bond of methanol. Therefore, at least two adjacent Pt sites are needed for methanol oxidation, irrespective of whether the reaction proceeds *via* the indirect or the direct pathway. By contrast, the direct pathway for formic acid oxidation has been reported to be catalysed even on single Pt atoms.

### Selective oxidation reaction of organic substrates

The aqueous electrochemical oxidation of organic substrates into valuable compounds has attracted much attention because it can be carried out under ambient conditions without the use of toxic or flammable reagents. The major challenge is that electrocatalysts with highly reactive species as active centres are required for the activation of robust C–H bonds in hydrocarbons. High-valency ruthenium-oxo species, such as Ru^IV^=O or Ru^V^=O,^16^ are one group of prospective catalysts to satisfy this requirement. However, in aqueous solutions, these metal-oxo species also serve as active centres for the OER based on water oxidation, which competes with C–H oxidation reactions.^5^ Thus, suppressing the OER is critical for the development of effective hydrocarbon oxidation catalysts for aqueous electrochemistry.

**Fig. 4:**
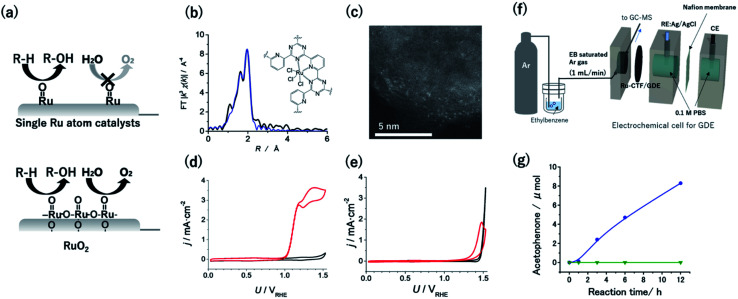
(a) Schematics of selective alcohol oxidation on single-Ru sites against the OER, as compared with oxidation on RuO_2_. (b) FT-EXAFS spectra and (inset) schematic structure of the Ru-CTF. Black and blue lines represent the measured and fitted results, respectively. (c) Representative HAADF-STEM image of the Ru-CTF. Plots of *j vs. U* for (d) the Ru-CTF and (e) RuO_2_ in 0.1 M HClO_4_ (pH 1) with (red line) and without (black line) 0.14 mM benzyl alcohol. (f) Schematic of the setup for electrochemical gaseous ethylbenzene oxidation by a GDE carrying a Ru-CTF. (g) The amount of acetophenone generated as a function of time at 1.5 V *vs.* RHE for (blue) the Ru-CTF/GDE and (green) the CTF/GDE with gaseous ethylbenzene. Reproduced in part with permission from ref. 56 and 58. Copyright 2017 the Royal Society of Chemistry and 2020 American Chemical Society, respectively.

Given that the OER is facilitated by the coupling of two neighbouring Ru=O species, singly isolated Ru atoms were expected to oxidize hydrocarbons selectively over the OER (Fig. 4a).^55^ Therefore, CTF supports were adopted to obtain single Ru catalytic sites. The Ru atoms in a Ru-CTF were confirmed using EXAFS (Fig. 4b) and HAADF-STEM images (Fig. 4c) to be singly isolated and anchored to the N atoms of the CTF, similar to the anchoring of Pt onto a CTF.^56^ In particular, the EXAFS spectra in Fig. 4b show peaks assignable to Ru–N and Ru–Cl bonds at 0.16 nm and 0.19 nm, respectively. By contrast, peaks corresponding to Ru–O–Ru bonds (0.28 nm) of RuO_2_ were not detected. Fig. 4d and e show the changes in current density (*j*) at different potentials (*U*) for the Ru-CTF and RuO_2_ electrodes in 0.1 M HClO_4_ solutions with and without 14 mM benzyl alcohol. In the absence of benzyl alcohol (black lines in Fig. 4d), the Ru-CTF generated almost no current in the examined potential regions, whereas the oxidation current associated with the OER started to flow at 1.4 V *vs.* RHE for RuO_2_.^56,57^ By contrast, the onset potential for the benzyl alcohol oxidation reaction on the Ru-CTF was 1.0 V *vs.* RHE (red lines in Fig. 4d), which is 200 mV more negative than the value associated with the RuO_2_ electrode (Fig. 4e). Thus, the Ru-CTF could effectively oxidize benzyl alcohol and showed almost no OER activity in any of the examined potential regions, whereas RuO_2_ oxidized water to O_2_.

In contrast to the inactivity of single Pt atoms toward alcohol oxidation reactions described in the previous section, single high-valency Ru can function as an active centre for the oxidation of organic substrates. High-valent metal-oxo species have been reported to facilitate net hydrogen atom abstraction and/or net oxygen atom insertion through a relatively weak interaction between metal centres and substrates.^56,57^ By contrast, the strong adsorption of substrates to activate C–H bonds is the first step in the oxidation of organic substrates on Pt or Pd surfaces.

High-valent Ru=O species formed on Ru-organic complexes have been demonstrated to oxidize not only alcohols but also hydrocarbons with more stable C–H bonds in organic electrolytes.^16^ However, green and cost-effective aqueous electrochemistry is ideal as a sustainable technology, although many raw organic substances are poorly soluble in aqueous solutions. Kato *et al.* realized hydrocarbon oxidation reactions in aqueous solutions using gas-diffusion electrodes (GDEs) with immobilized Ru-CTFs. Ar gas saturated with ethylbenzene (1.2 × 10^3^ Pa) was supplied from one side of the GDE as a model substrate (Fig. 4f).^58^ When a potential of 1.5 V *vs.* RHE was applied to the GDE electrode, acetophenone was confirmed to be selectively generated as the product of ethylbenzene oxidation without other detectable products. The concentration of generated acetophenone reached 8.3 μmol after 12 h of electrolysis (blue points in Fig. 4g).^58^ By contrast, the generated acetophenone was almost negligible when a bare CTF (*i.e.*, without Ru) was used (green points in Fig. 4g). These results indicate that the Ru atoms in the CTF served as active centres for ethylbenzene oxidation. The oxygen-atom insertion by Ru=O into the C–H bond of a methyl group was selectively facilitated, resulting in selective acetophenone generation. This report was the first demonstration that single-atom electrocatalysts can catalyse electrochemical hydrocarbon oxidation reactions in aqueous electrolytes.^58^ Notably, the degradation of the ethylbenzene oxidation activity of the Ru-CTF was negligible even after 48 h of electrolysis. This stability is much better than that of the corresponding Ru-based organometallics.^58^

### Selective CO_2_ reduction reaction *vs.* hydrogen evolution

Excessive emission of CO_2_ from the use of fossil fuels is becoming a serious issue for the sustainable development of our society. Thus, developing technologies that use CO_2_ as an alternative carbon feedstock and transform it into valuable chemicals, thereby creating a closed carbon cycle, is important. Electrochemical CO_2_ reduction has attracted intensive attention because it can be carried out using electricity generated from renewable energy sources directly. Two kinds of selectivity are necessary for the efficient electrochemical CRR: (1) the HER must be suppressed because it competes with the CRR under the range of operating potentials (substrate selectivity) and (2) higher-value-added products, such as C2 and C3 compounds or liquid fuels, should be produced (product selectivity).

We here review the substrate selectivity toward CO_2_ and against H^+^ (*i.e.*, the CRR *vs.* the HER). Although Cu bulk metal produces organics with relatively high selectivity during the CRR, it still exhibits greater than 30% faradaic efficiency (FE) for the HER.^59,60^ In addition to bulk metals, organic complexes are an alternative class of electrocatalysts for the CRR. Fe- or Co-based N4 macrocycles, in particular, have been reported to efficiently catalyse the reduction of CO_2_ to CO and methane.^61,62^ On the basis of these reports, Lin *et al.* synthesized new COFs comprising Co porphyrin units as CO evolution catalysts.^63^ In contrast to these Co–N4 compounds, Cu- or Ni-macrocycles are known to exhibit poor activity toward CO generation from CO_2_ because they weakly bind COOH, the key intermediate for CO.^61,64^ Thus, the choice of an appropriate metal species for electrocatalytic reactions from the viewpoint of adsorption strength of the substrate and/or intermediates is clearly important. In addition to metal species, another important factor influencing the adsorption strength is the coordination structure, especially the coordination number (CN) of the metal centres. Though coordinatively unsaturated metals are mostly unstable, the rigid framework of a COF would stabilize open coordination single-metal sites. Iwase *et al.* recently theoretically demonstrated that metal centres with a lower CN generally adsorb ORR intermediates more strongly when CTFs are used as SAC supports because of low steric hindrance and many accessible d-orbitals.^41^ Therefore, CTFs may improve the catalytic performance of metal species previously thought to exhibit no CO_2_ reduction activity (*e.g.*, Ni or Cu) by increasing the COOH adsorption strength.

**Fig. 5:**
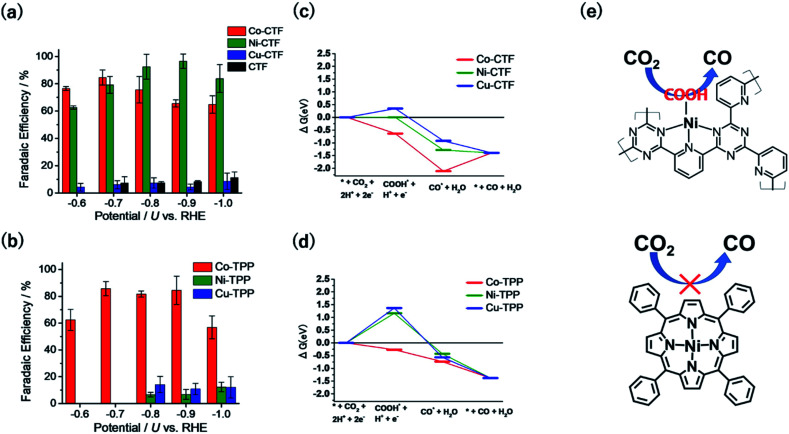
FE for CO production on (a) M-CTFs and (b) M-TPPs in CO_2_-saturated KHCO_3_ electrolyte. Free-energy diagrams for each reaction coordinate for CO generation for (c) M-CTFs and (d) M-TPPs at −0.87 V *vs.* computational hydrogen electrode. (e) Schematic of the Ni-CTF unsaturated coordination sites that strongly bind the COOH intermediate and generate CO (top), compared with the Ni-TPP (bottom). Reproduced from ref. 65 under the CC BY 3.0 license.

Fig. 5a and b show the FEs of CO for the M-CTF and M-tetraphenylporphyrins (M-TPPs, M = Co, Ni, Cu), respectively. Among the investigated M-TPPs, the Co-TPP exhibited CO generation activity, whereas the Co-CTF and Ni-CTF both efficiently reduced CO_2_ to CO.^65,66^ The FE of CO for the Ni-CTF at −0.8 to −0.9 V exceeded 90%. EXAFS analyses showed that the CNs of Co-, Ni-, and Cu-CTFs were 3.2, 3.4, and 3.4, respectively.^40,67^ Thus, compared with the corresponding M-N4 compounds, the M-CTFs have an unsaturated coordination structure. Free-energy diagrams of CO production for M-CTFs and M-TPPs were calculated using DFT (Fig. 5c and d).^65^ All elementary steps on the Co-TPP are exergonic, whereas the formation of adsorbed COOH is endothermic for the Ni- or Cu-TPPs. In contrast to the M-TPPs, the Ni-CTF exhibited a downhill pathway for all of the elementary steps, consistent with its high CRR selectivity against the HER. The open-coordination sites of metal centres in M-CTFs more strongly bind COOH than the corresponding coordinatively saturated metal centres in M-TPPs (Fig. 5e). Yan *et al.* also synthesized single Ni sites with an unsaturated coordination structure in porous carbon and demonstrated that the Ni site exhibited optimal OH adsorption strength and high CO production activity.^68^ Thus, the choice of an SAC with an appropriate CN can lead to a drastic improvement in the electrocatalytic activity of metal species previously thought to exhibit poor activity. Table 1 summarizes recent reports in which Ni-SACs were used as CRR catalysts. The Ni-CTF showed one of the highest FEs reported for CO production. In addition, when the catalyst was supported on GDEs, CO_2_ was reduced to CO with a high current density (over 230 mA cm^−2^), indicating that the Ni-CTF exhibits a high turnover frequency for the CRR.

**Table 1:** Summary of Ni-based SACs for CO_2_ reduction reactions

Catalysts	Catalyst loading [mg cm^−2^]	Electrolyte	Potential [*vs.* RHE]	FE for CO	*j*_CO_ [mA cm^−1−2^]	Ref.
Ni–N-graphene	0.3	0.1 M KHCO_3_	−0.8	90%	1.5	64
Ni_2_-CPD_Py_973	0.06	0.1 M KHCO_3_	−0.8	94%	0.34	69
Ni–N_4_–C	0.06	0.1 M KHCO_3_	−0.8	92%	0.5	70
Ni–N-MEGO	0.5	0.5 M KHCO_3_	−0.7	92%	26.8	71
Ni-NCNT	0.8	0.5 M KHCO_3_	−0.75	92%	22	72
CNT-fiber supported Ni-SA	3.5	0.5 M KHCO_3_	−1.0	97%	48.7	73
Ni–N-rGO	0.2	0.5 M KHCO_3_	−1.0	97% (at −0.8 V)	42	74
Ni-SA-NCs/MEA	0.3	0.5 M KHCO_3_	—	96%	380	75
Ni-CTF	0.3	0.1 M KHCO_3_	−0.9	97%	1.8	65
Ni-CTF/GDE	0.4	1 M KOH	—	78%	234	66
Ni-CTF/GDE	0.4	0.01 M HClO_4_/0.1 M NaClO_4_ (pH = 2)	—	65%	1.9	66

On the basis of the Sabatier principle, optimal electrocatalysts have moderate binding strength with reaction intermediates not only for the CRR but for many other reactions.^76^ Metal species and their CNs strongly affect the adsorption strength.^77,78^ Given that a wide variety of metal centres (from 3d to 5d metals) can be doped into the micropores of COFs and that their CNs can also be tuned by choosing appropriate monomers, COFs are ideal supports that enable the adsorption strength to be adjusted to optimal values.^41^

### Selective CO_2_ reduction reaction to organics

We next discuss the product selectivity for the CRR on SACs. In 1986, Hori *et al.* reported that Cu metal electrodes efficiently catalysed the reduction of CO_2_ to organics, including C1–C3 compounds (methane, ethylene, and alcohols).^59,60,79^ If two CO molecules adsorbed onto adjacent metal sites are assumed to dimerize to C2 or longer carbon chains, then single-metal sites should produce only C1 compounds. For example, transition metals (Sc, Ti, V, and Fe)–phthalocyanine monolayers or single atoms (Pt or Ir) supported on graphene or TiC have been predicted on the basis of first-principles calculations to produce methane or methanol.^80–82^ Unfortunately, however, the experimentally observed CRR product generated by SACs has only been CO in most of the related CRR studies, as typified by the aforementioned examples, with the exception of the Cu-based catalysts. In the case of Cu-based catalysts, the operating potential for the CRR is usually more negative than −0.6 V, and single Cu sites tend to agglomerate to form metal nanoparticles under these cathodic conditions.^83–85^ For example, Weng *et al.* demonstrated that Cu nanoclusters were formed from Cu(ii)–phthalocyanine during electrolysis and served as the active centre for hydrocarbon evolution reactions *via* the CRR.^84^ Therefore, demonstrating that single Cu sites are active centres to produce organics is difficult. SACs composed of non-Cu metals for converting CO_2_ to organics are therefore introduced here.

In 2015, Varela *et al.* demonstrated that single-metal-doped carbons reduced CO_2_ not only to CO but also to hydrocarbons. In particular, Fe or Fe–Mn co-doped carbons produced methane at −0.9 V *vs.* RHE.^86,87^ First-principles calculations and experimental studies have shown that two pathways exist for methane generation on single-site Fe–N–C catalysts: the proton-decoupled electron transfer pathway (CH_2_O as a solvated intermediate) and the proton-decoupled electron transfer pathway (no releasing intermediate).^88^ Shen *et al.* reported that an immobilized Co protoporphyrin on a pyrolytic graphite electrode reduced CO_2_ to methane and CO in an aqueous acidic solution.^89,90^ Specifically, the methane production on Co–protoporphyrin was facilitated at pH 1, and the intermediate was HCOH. However, the FEs for methane were only approximately 1–2% in both cases of Fe- and Co-SACs. Thus, an improvement of hydrocarbon selectivity is strongly demanded. This goal can potentially be achieved through precise tuning of the CO adsorption strength on SACs. The design flexibility of COFs as a result of the abundance of monomers might enable the modulation of CO adsorption, resulting in the production of hydrocarbons.

### Selective reduction reaction of nitrogen oxides to form N–N bonds

**Fig. 6:**
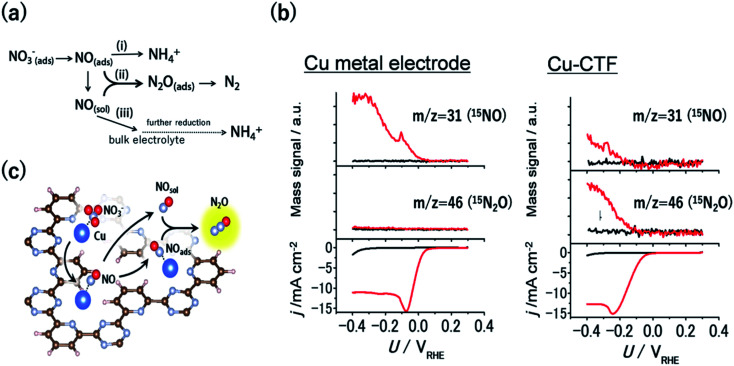
(a) General scheme for nitrate reduction under acidic conditions (b) mass signals for ^15^NO and ^15^N_2_O and the corresponding *j*–*U* curve of (left) Cu-metal and the (right)Cu-CTF in 0.1 M HClO_4_ (red) with and (black) without 0.1 M Na^15^NO_3_. (c) Schematic of the reaction mechanism for N_2_O formation on the single-Cu site catalyst (Cu-CTF). Reproduced in part with permission from ref. 94. Copyright 2016 American Chemical Society, respectively.

Nitrate contamination due to industrial drainage and overfertilization is becoming an important issue, and the denitrification of nitrate-containing water is an urgent need to sustain the global nitrogen cycle. One of the promising approaches to denitrification is the electrochemical reduction of nitrate.^3^ An electrochemical method can be conducted even for highly acidic solutions or in the presence of a high concentration of nitrate, where current biological treatments cannot be utilized. The electrochemical nitrate reduction reaction (NO_3_RR) in acidic electrolytes occurs *via* a stepwise mechanism (Fig. 6a). Considering this reaction mechanism, the reductive dimerization of NO to N_2_O (path ii in Fig. 6a) is essential for the formation of dinitrogen, which is a desired denitrification product. The formation of N_2_O is known to be facilitated *via* the Eley–Rideal-type mechanism, whereby solvated NO reacts with surface-bound NO.^91,92^ Therefore, the balance between the amount of surface-bound NO and the amount of NO released from electrodes is important for the dimerization, indicating that optimal NO_3_RR electrocatalysts would have moderate binding energy with NO. For example, Pt bulk electrodes, which bind NO too strongly, reduce the adsorbed NO to NH_4_^+^ ions on the surface (path i in Fig. 6a). Conversely, although Cu bulk metal is the most active surface for the NO_3_RR among non-noble-metal electrodes, NO molecules generated through the reaction diffuse into an electrolyte because of the weak adsorption of NO onto Cu (path iii in Fig. 6a).^93^

As mentioned in the previous section, the 3d atoms of M-CTFs possess unsaturated first coordination, resulting in open coordination sites and low steric hindrance, which in turn results in the strong adsorption of intermediates. Thus, the Cu-CTF is expected to bind even more strongly to NO than Cu bulk metal. Fig. 6b shows the results of the electrochemical mass spectrometry (ECMS) analysis of the volatile products generated during the NO_3_RR in 0.1 M HClO_4_ with 0.1 M Na^15^NO_3_ by Cu bulk metal and by the Cu-CTF. In the case of Cu metal electrodes, only the ^15^NO mass signal traced the *j vs. U* characteristic. By contrast, for the Cu-CTF, not only the mass signal for ^15^NO but also that for ^15^N_2_O increased from the onset of the NO_3_RR current.^94^ The FE for N_2_O on the Cu-CTF was as high as 75% at −0.2 V *vs.* RHE with 1 M nitrate, whereas the N_2_O formation on Cu metal was almost negligible (<0.5%).^94^ These results indicate that the single Cu sites of the Cu-CTF efficiently catalysed the formation of N_2_O (*i.e.*, N–N bond formations). The NO adsorption energy on Pt(111), Cu(111), and the Cu-CTF, as calculated using DFT, was 251, 77.5, and 140 kJ mol^−1^, respectively. As expected on the basis of the aforementioned results, the Δ*E*_NO_ of the Cu-CTF was intermediate, between those of Pt(111) and Cu(111), which indicates that the Cu-CTF has the optimal NO binding strength (Fig. 6c).^94,95^

**Fig. 7:**
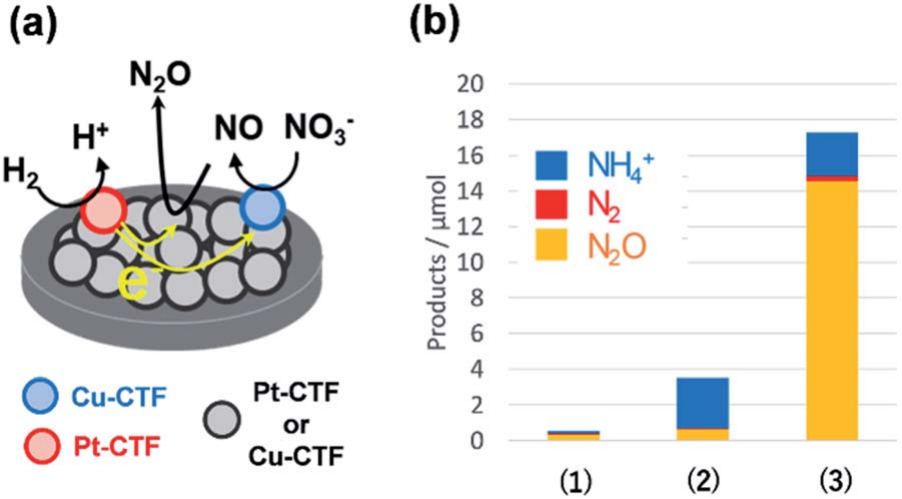
(a) Local-cell catalyst composed of the Cu-CTF and Pt-CTF. (b) NO_3_RR yields on (1) the Cu-CTF, (2) Pt-CTF, and (3) Cu-CTF and Pt-CTF (the local-cell catalyst) in 0.1 M HClO_4_ with 0.1 M Na^15^NO_3_ under H_2_ gas. Reproduced in part with permission from ref. 99. Copyright 2018 American Chemical Society.

By contrast, single Pt atoms supported on the CTF are almost inactive toward the NO_3_RR, although bulk Pt electrodes are known to effectively convert nitrate to ammonia.^96^ This unique inactivity of single Pt atoms for the NO_3_RR is explained as follows. Under-potentially deposited hydrogen (upd-H) is not observed on single Pt atoms because upd-H is formed on Pt ensemble sites, such as steps, hollows, defects, and three-fold sites.^96^ The first step of the NO_3_RR on Pt electrodes (NO_3_^−^ to HNO_2_) is known to occur through the reaction among adsorbed hydrogen and adsorbed nitrate *via* a Langmuir–Hinshelwood mechanism.^97,98^ Therefore, single Pt atoms exhibit negligible NO_3_RR activity because of a lack of adsorbed protons. Specifically, the Pt-CTF and Cu-CTF can serve as selective electrocatalysts (half-cell catalysts) for the HOR (for details, see the previous subsection) and the NO_3_RR to N_2_O even in the presence of both nitrate and hydrogen, respectively.^99,100^ Given these selective half-cell reactions, we successfully developed a novel system for the selective reduction of nitrate to N_2_O using H_2_. The system is based on the principle of a local cell in which the CTF-based catalysts promoting the two half-cell reactions are electrochemically connected *via* a conductive plate.^99,100^ The glassy carbon plate modified with only the Cu-CTF, with only the Pt-CTF, or with both Cu-CTF and Pt-CTF (Fig. 7a) was immersed in H_2_-saturated 0.1 M HClO_4_ solutions containing 0.1 M nitrate. Fig. 7b shows that the reaction products of the Cu-CTF were only small quantities of nitrogen compounds.^99^ The Pt-CTF generated a small amount of product primarily consisting of NH_3_. In the case of the specimen containing both Pt-CTF and Cu-CTF, the production of N_2_O was substantially increased. These results indicate that a local-cell process resulting from the coupling of the HOR and NO_3_RR occurred on this device.

## Stability of single-metal atoms doped in CTFs

Here, let us consider the stability of single-3d-metal-atoms-doped CTFs based on experimental and theoretical results. The stabilization energies for single-3d metal atoms in COFs with bipyridine (CN = 2), terpyridine (CN = 3), and porphyrin (CN = 4) moieties have been calculated using DFT. As predicted, lower-coordination metals are less stable. In the case of Ni-SACs, the stabilization energies for the Ni atoms with Ni–N4, Ni–N3 and Ni–N2 were 8.9 eV, 3.2 eV and 2.7 eV, respectively. Notably, 3d metal atoms in COFs with a low CN (CN = 2, 3) are obviously more stable than metal atoms immobilized on pristine graphene as a reference.^41^ An electrocatalytic durability test of the Ni-CTF for the CRR revealed that the decrease in FE for CO production was almost negligible during 3 h of electrolysis, whereas the cathodic current slowly decreased.^65^ In addition, a single-Cu-atom-doped CTF displayed greater stability than a Cu-based molecular complex as the ORR catalyst in neutral solutions because of the densely cross-linked structure formed by covalent bonds.^67^ (Details of the ORR by the Cu-CTF are outside the scope of the present paper because the ORR on the Cu-CTF is not a selective reaction.) However, the stability of M-CTFs is insufficient for their actual application, and CTFs are unfortunately more fragile than inorganic supports. Further studies on improving the stability by choosing appropriate monomers and coordination structures are required for future implementation.

## Conclusions

In the present review, we introduced the selective electrocatalytic properties of SACs, with particular focus on triazine-based COFs as supports. CTFs can immobilize a high number of Pt-group metals because of the coordination bonds with abundant N atoms in their pores. Pt-group SACs exhibit poor activity for the several reactions facilitated by the corresponding bulk metals, such as the ORR and the NO_3_RR, because of the absence of neighbouring metal sites. The unique substrate selectivity of Pt-group SACs enables us to use substrates with contaminants (*i.e.*, low-purity substrates). In the case of a single-3d-metal-doped CTF, coordinatively unsaturated metal centres are stabilized by the rigid frameworks, and they strongly bind substrates and/or intermediates. The adsorption strength of intermediates is enhanced by the open-coordination sites, resulting in various unique electrocatalytic selectivities. On the basis of this review, we expect that single-atom sites with a defined coordination structure in COFs will become a novel platform for selective electrocatalysts for applications intended to solve energy and environmental issues. Notably, the unique selectivities of SACs in this review, such as the oxygen-tolerant HOR on single Pt atoms, the selective reduction of nitrate, and the oxidation of organics against the OER on single Ru atoms have been demonstrated only using M-CTFs as catalysts. We expect that the selectivity of SACs can be further improved through the use of other sophisticated supports.

One next challenging task is to increase the density of SACs on supports. Pennycook *et al.* successfully doped a high number of Cu single atoms coordinated with N atoms using porous carbons as the substrate.^39^ The Co–N-doped porous carbons efficiently catalyzed the dinitrogen reduction to ammonia because of the high population of single Co atoms in the pore. Therefore, the effective use of the microporosity of CTFs is one approach to overcoming this issue. Though metal atoms are now doped into COFs *via* a simple impregnation method, more sophisticated metal doping methods, such as atomic layer deposition and chemical vapour deposition, may enable us to use the inner pores of COFs more effectively and to increase the density of single-atom sites.

In addition to catalytic activity, catalytic selectivity and cost, compatibility with mass production methods is a basic requirement for practical catalysts. Compared with studies on nanocarbons or other inorganic materials, studies on the mass production of COFs are still lacking. The development of novel scalable synthetic methods under mild reaction conditions is a key future research target.

## Conflicts of interest

There are no conflicts to declare.
